# Microbial Dynamics of a Specialty Italian Raw Ewe’s Milk Cheese Curdled with Extracts from Spontaneous and Cultivated *Onopordum tauricum* Willd

**DOI:** 10.3390/microorganisms11010219

**Published:** 2023-01-15

**Authors:** Giorgia Rampanti, Luca Belleggia, Federica Cardinali, Vesna Milanović, Andrea Osimani, Cristiana Garofalo, Ilario Ferrocino, Lucia Aquilanti

**Affiliations:** 1 Dipartimento di Scienze Agrarie, Alimentari ed Ambientali, Università Politecnica delle Marche, via Brecce Bianche, 60131 Ancona, Italy; 2 Department of Agricultural, Forest, and Food Science, University of Turin, Largo Paolo Braccini 2, 10095 Grugliasco, Italy

**Keywords:** Caciofiore cheese, vegetable rennet, thistle rennet, *Onopordum tauricum* Willd., microbial dynamics, Illumina DNA sequencing

## Abstract

Milk coagulants prepared by maceration of flowers harvested from both spontaneous and cultivated *Onopordum tauricum* Willd. and a commercially available coagulant obtained from *Cynara cardunculus* L. (control) were assayed for small-scale manufacturing of Caciofiore, an Italian specialty raw ewe’s milk cheese produced in a family run dairy farm located in the Marche region (Central Italy). The microbiota of the three thistle-based milk coagulants and their effect on the microbial dynamics of raw milk cheeses during fermentation and maturation (from day 0 up until day 60) were investigated through a combined approach based on viable counting and Illumina DNA sequencing. In both the control and experimental cheeses, despite the slight differences emerged depending on the coagulant used, *Lactococcus lactis* and *Debaryomyces hansenii* were the prevalent species among bacteria and fungi, respectively. Moreover, raw ewe’s milk was the main factor affecting the evolution of both the bacterial and fungal microbiota in all cheeses. The overall similarities between control and experimental cheeses herein analyzed supports the exploitation of *Onopordum tauricum* Willd. as an alternative milk coagulating agent for production of Caciofiore and, more in general, raw ewe’s milk cheeses.

## 1. Introduction

Cheese is a complex food, whose characteristics largely depend on basic (milk, salt, starter culture, and rennet) and optional unconventional ingredients (e.g., grated lemon peel, aromatic herbs, etc.), as well as on the parameters applied during cheesemaking, which, in turn, affect the biochemical and microbiological transformations occurring during fermentation and ripening [1,2].

It is estimated that more than a thousand varieties of cheese are produced in the world by combining the aforementioned factors [3]; for approximately 75% of produced cheeses, coagulation is achieved through the addition of rennet to the milk [4].

Historically, the term “rennet” refers to the crude extract from bovine calf stomach containing a complex set of protease enzymes that curdles the casein [5]. However, starting from the 1950s, due to a shortage in the supply of animal rennet, the search for “rennet substitutes” began. Four main types of milk coagulants are currently available, being animal rennet, microbial rennet, fermentation-produced chymosin, and vegetable rennet [6].

Concerning the latter, the ability of some plants to coagulate milk has been known since ancient times; in his treatise *De Re Rustica* (ca. 50 AD), Lucius Columella was the first to suggest the use of wild thistle flowers, safflower seeds, and fig latex for milk coagulation. In the past decades, the list of vegetable sources of milk-clotting enzymes has increased; *Fœniculum vulgare* [7], *Solanum elaeagnifolium* [8], *Morinda citrifolia* L. [9], *Solanum tuberosum* [10], and *Salpichroa origanifolia* [11] are just a few examples that have recently been reported in the literature. 

Nevertheless, thistle flowers are still given particular attention [12], especially in Mediterranean countries, such as Italy, Portugal, and Spain, where these plants grow spontaneously and are traditionally exploited for manufacturing of typical ovine cheeses [13,14,15,16,17]. 

The term “thistles” indicates plants belonging to several genera within the Asteraceae family, including *Carduus*, *Cirsium*, *Onopordum*, *Carlina, Scolymus*, *Silybum*, and *Cynara*. *Cynara cardunculus* L. is undoubtedly the most investigated source of milk clotting enzymes [18,19,20,21,22]; however, this is not the only thistle species with a milk coagulating potential [23]. 

Very recently, *Onopordum tauricum* Willd. has been deeply investigated for its curdling properties towards ewe’s, goat’s, and cow’s milk [24,25]. Native to Southern Europe and Southwestern Asia, this thistle grows spontaneously in Italy, especially in marginal areas of Central and Southern regions [26]. 

Commonly known as Taurian thistle or Bull cottonthistle, *O. tauricum* owes its name to the Greek term ονο, onos meaning donkey, and πορδη, pordè meaning fart, for the alleged effects of intestinal turbulence that the plant causes to donkeys who are greedy for it. *O. tauricum* is a vigorous biennial, or short-lived perennial with coarse, spiny leaves and conspicuous spiny winged stems. The plants are 100–300 cm in height, highly branched, with broad leaves with longitudinal spines and mostly singular red-purple flowers at the terminals of the main and side stems. The bright purple flower heads are 3 to 4 inches in diameter. The heads consist of numerous spiny-tipped bracts resembling an artichoke before the bud opens. Due to its growth habits and appearance, very close to cotton thistle (*Onopordum acanthium* L.), *O. tauricum* is worldwide considered a weed; in fact, with its dense stands, it can crowd out native vegetation, and hence reduce forage availability and quality as well as render riparian areas and pasture impenetrable for medium to large animals [27].

Though indicated as a source of prebiotics and antioxidants [28,29], and recognized as a promising crop in terms of adaptation capacity, required inputs, and yields [30,31,32], this species is currently still underused. 

Specialty cheeses are cheeses often made in a limited number or smaller batches, with special attention paid by cheesemakers to flavor and texture. At this regard, rennet is recognized as a source of both enzymes and microorganisms, with a potentially high impact on quality and texture of final cheeses. However, to date, most of the available studies on thistle rennet address technological and chemical properties of these milk coagulants, while less data is available on their microbiological properties and those of thistle-curdled cheeses [13,14,15,17,20,33,34,35]. 

In the present study, the microbiota of two milk coagulants prepared by maceration of flowers from both spontaneous and cultivated *Onopordum tauricum* Willd., and of a commercially available rennet obtained from *Cynara cardunculus* L. was investigated through a combined approach based on viable counting and Illumina DNA sequencing. In addition, the impact of these coagulants on the microbial dynamics of small-scale manufactures of Caciofiore a specialty raw ewe’s milk cheese produced in a family run dairy farm located in the Marche region (Central Italy) was also investigated using the same analytical approach. 

## 2. Materials and Methods

### 2.1. Preparation of Crude Extracts (CE)

**Figure 1:**
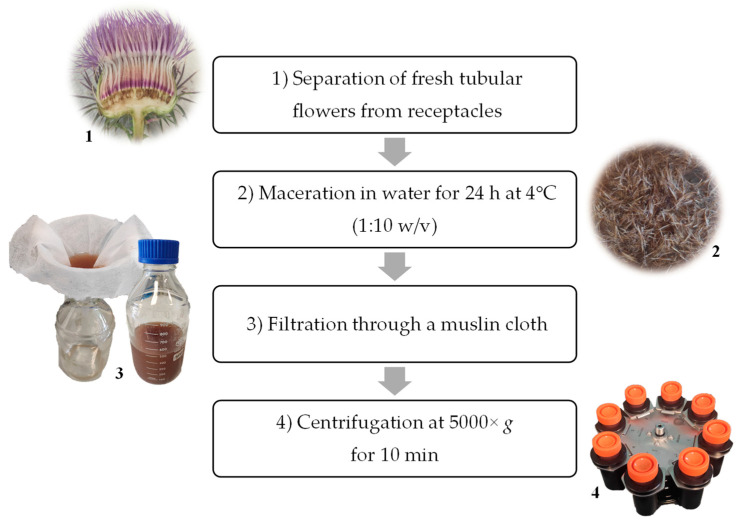
Flow chart illustrating the followed steps for the preparation of crude extracts (CE) from *Onopordum tauricum* Willd. flowers heads.

Flower heads from spontaneous *O. tauricum* Willd. plants were collected in July 2020 in the outer fringes of the Monti Sibillini National Park in the municipality of Visso (MC, Italy) according to Foligni et al. [25], while flower heads from cultivated plants were harvested in July 2020 from the experimental and didactic farm “Pasquale Rosati” (Università Politecnica delle Marche, Agugliano, AN, Italy) according to Zenobi et al. [31]. The crude extracts (CE) from spontaneous (st) and cultivated (ct) flower heads were prepared following the procedure outlined by Mozzon et al. [24] and illustrated in Figure 1. Briefly, the purple tubular flowers were separated from the receptacle and macerated in demineralized water (1:10 *w*/*v*) for 24 h at 4 °C; the resulting aqueous extracts were cleared by filtration through a muslin cloth and subsequent centrifugation (5000× *g*). Finally, the crude extracts were freeze-dried (VirTis Advantage benchtop freeze dryer, Steroglass S.r.l., Perugia, Italy), stored at −20 °C, and reconstituted in demineralized water at the time of use.

### 2.2. Cheesemaking and Sampling

**Figure 2:**
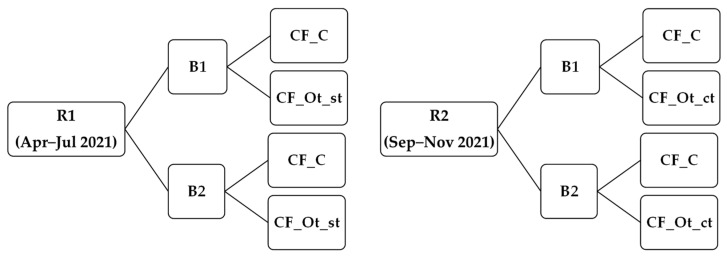
Experimental plan of the cheesemaking trials. Two production rounds were run, the first (R1) between April and July 2021, and the second (R2) between September and November 2021; for each Round, trials were repeated on two different days (Batch 1, B1, and Batch 2, B2, respectively). On the same day, both control (CF_C) and experimental (CF_Ot_st in R1 and CF_Ot_ct in R2) Caciofiore cheeses were produced.

Cheesemaking trials for manufacturing of Caciofiore (CF) were conducted in a family run dairy farm located in Pieve Torina (MC), in the Monti Sibillini National Park (Marche Region, Italy), following a traditional method (Supplementary Figure S1) and using raw *Sopravissana* breed ewe’s milk, without the addition of starter cultures. The experimental plan for the cheesemaking trials is depicted in Figure 2. In the first production round (R1), conducted between April and July 2021, experimental cheeses (coded as CF_Ot_st) were curdled with the reconstituted CE_st, while in the second production round (R2), conducted between September and November 2021, experimental cheeses (coded as CF_Ot_ct) were curdled with the reconstituted CE_ct. 

For each round, cheesemaking trials were repeated twice, on two different days, using two different batches of ewe’s raw milk (B1 and B2, respectively). For each round, control cheeses (coded as CF_C) were also produced using a commercial vegetable coagulant (Dairen, Dairy and Food, Castel San Pietro Terme, BO, Italy) obtained from flowers of *Cynara cardunculus* L. (Cc). For each cheese manufacture, seven cheese wheels, each weighing about 250 g, were produced. Both control (CF_C) and experimental cheeses (CF_Ot_st and CF_Ot_ct) were ripened for 60 days at 12 °C and 70% of relative humidity (RH). 

### 2.3. Physico-Chemical Measurements

The pH values of milk and Caciofiore cheese samples collected immediately after molding (day 0) as well as after 2, 5, 15, 30, 45, and 60 days of ripening were determined using a pH-meter Model 300 (Hanna Instruments, Padova, Italy) equipped with a solid electrode. Total titratable acidity (TTA) was determined on 10 g of sample homogenized in 90 mL of distilled water, titrated to a pH value of 8.3 with a 0.1 N solution of NaOH [36]. Percentage (%) TTA of lactic acid equivalents was calculated according to the following formula:

*volume of titrant* × *N* × *90*
TTA = ----------------------------------- × 100
*weight of sample* × *1000*

where *N* is the normality of the titrant and 90 is the equivalent weight for lactic acid.

Both pH and TTA were reported as mean ± standard deviation of two biological and two technical replicates.

### 2.4. Microbiological Analyses

Microbial counts were determined on commercial (Cc) and experimental coagulants (reconstituted CE_st and CE_ct), raw milk, and Caciofiore cheese samples; 10 g of each cheese sample was homogenized with 90 mL of sterile peptone water (bacteriological peptone, 1 g L^−1^, Oxoid, Basingstoke, UK) using a stomacher apparatus (400 Circulator, International PBI, Milan, Italy) for 2 min at 260 rpm [37]. Thistle-based coagulants, raw milk, and cheese homogenates were serially diluted in sterile peptone water. Aliquots of decimal dilutions were inoculated in duplicate on the opportune growth media for the enumeration of the following microbial groups: (i) total mesophilic aerobes on Plate Count Agar (PCA) incubated at 30 °C for 48 h; (ii) presumptive lactobacilli on de Man, Rogosa, Sharp (MRS) agar incubated at 30 °C for 48 h; (iii) presumptive mesophilic and thermophilic lactococci on M17 agar incubated at 22 and 45 °C, respectively, for 48 h; (iv) Enterobacteriaceae on Violet Red Bile Glucose Agar (VRBGA) incubated at 37 °C for 24 h; (v) coliforms and *Escherichia coli* on Chromogenic Coliform Agar (CCA) incubated at 37 °C for 24 h; (vi) Pseudomonadaceae on Pseudomonas Agar Base (PAB) supplemented with Cetrimide, Fusidic Acid, and Cephaloridine (CFC), incubated at 30 °C for 48 h; (vii) yeasts and molds on Rose Bengal (RB) agar incubated at 25 °C for 72 h. The results of viable counts were expressed as the log of colony-forming units (cfu) per gram or mL of sample and reported as mean value ± standard deviation of two biological and two technical replicates.

### 2.5. DNA Extraction and Sequencing

The total microbial DNA was extracted from the cell pellets obtained by centrifugation of 1.5 mL of each biological replicate (dilutions 10^−1^) using the E.Z.N.A. soil DNA kit (Omega Bio-tek, Norcross, GA, USA). For each milk and cheese sample, the DNA extracts obtained from each biological replicate were pooled to reduce the inter-sample variability, as previously elucidated [38]. DNAs were quantified using the QUBIT dsDNA Assay kit (Life Technologies, Milan, Italy), and then diluted to 5 ng μL^−1^ to perform the metataxonomic amplicon sequencing. The amplification of the V3-V4 region of the 16S rRNA gene was carried out to determine the bacterial biota, in accordance with the conditions described by Klindworth et al. [39], whereas fungi were analyzed by amplifying the D1-D2 region of the 26S rRNA gene [40]. The amplicons were purified, tagged, and pooled following the Illumina standard protocol. Paired-end reads (2 × 250 bp) were sequenced by using a MiSeq platform (Illumina, San Diego, CA, USA) with v2 chemistry, according to the manufacturer’s instructions.

### 2.6. Metataxonomic Analyses

QIIME2 software was used to import and analyze raw reads (FASTQ format) [41]. Barcode adapters and primers were first removed, and sequences were quality filtered applying DADA2 algorithm to obtain amplicon sequence variants (ASVs) [42]. The taxonomic assignment of the bacterial ASVs was executed using the Greengenes 16S rRNA database, whereas the manually build database described by Mota-Gutierrez et al. [43] was used for the taxonomic assignment of the fungal ASVs. The resulting ASVs with a relative frequency > 1.0% in at least two samples were retained. To confirm the taxonomic assignment of both bacterial and fungal ASVs, a double check on the Basic Local Alignment Search Tool (BLAST) was performed. The results of metataxonomic analysis were deposited in the Sequence Read Archive of the National Center for Biotechnology Information (NCBI) under the bioproject accession number PRJNA843917.

### 2.7. Statistical Analysis

The Tukey–Kramer’s Honest Significant Difference (HSD) test (α = 0.05) was carried out to evaluate differences within thistle coagulants (Cc, CE_st, CE_ct), and cheese samples (CF_C vs CF_Ot_st and CF_C vs CF_Ot_ct) by one-way analysis of variance (ANOVA) using the software JMP^®^ Version 11.0.0 (SAS Institute Inc., Cary, NC, USA). The QIIME2 diversity plugin was used to calculate Alpha and Beta diversity indices for bacterial biota and mycobiota of thistle coagulants, raw milk, and Caciofiore cheese samples. Significant statistical differences (*p*-value < 0.05) were evaluated by the permutational multivariate analysis of variance (PERMANOVA). Principal component analysis (PCA) was performed on ASV relative frequencies using ade4 package in R v4.2.0. Differences in bacterial biota and mycobiota of Caciofiore cheese samples were evaluated by using the Kruskal–Wallis’s test (*p*-value < 0.05); the ggplot2 and reshape2 packages in the R environment were applied to obtain boxplot showing the relative frequencies of differential ASVs.

## 3. Results

### 3.1. pH, TTA, and Viable Counting

**Figure 3:**
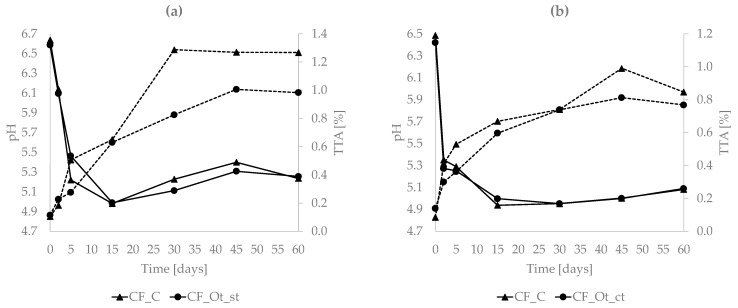
Results of pH and TTA in round 1 (**a**) and round 2 (**b**). pH values are indicated in the primary axis and the results represented with a continuous line; in the secondary axis, TTA values are indicated, and results represented with a dashed line.

Raw ewe’s milk used in the two cheesemaking rounds, R1 and R2, showed pH and TTA values of 6.74 ± 0.02 and 6.56 ± 0.02, respectively, for the first parameter, and of 0.15 ± 0.01 and 0.16 ± 0.02%, respectively, for the latter. The trends of pH and TTA in the cheeses during ripening are shown in Figure 3. In both rounds, a rapid drop in pH was observed during the first 5 days in all the samples; the minimum value was measured after 15 days, followed by a slight increase in the next ripening phase (30–60 days). An opposite trend was observed for TTA, but with a slower increase (maximum values reached between 30 and 45 days). No significant differences were observed between control and experimental cheese samples collected from the same round and the same sampling time (CF_C vs CF_Ot_st and CF_C vs CF_Ot_ct, respectively).

Table 1 shows the viable counts of milk coagulants. The commercial coagulant (Cc) showed counts < 1 Log cfu g^−1^ for all the microbial groups, except for yeast (6.36 ± 0.02 Log cfu g^−1^). The load of presumptive lactobacilli and *Escherichia coli* was <1 Log cfu g^−1^ for all coagulants. The two experimental coagulants from flowers of spontaneous (CE_st) and cultivated (CE_ct) *O. tauricum* were characterized by similar counts of molds, whereas significant differences of about 1–1.5 order of magnitude were observed in the loads of total mesophilic aerobes, presumptive lactococci, and presumptive thermophilic cocci, with higher values in CE_st compared to CE_ct. Even greater differences emerged in the viable counts of the remaining microbial groups, with CE_st showing counts of 3.04 ± 0.06, 3.51 ± 0.16, 3.64 ± 0.11, and 2.90 ± 0.00 Log cfu g^−1^ for Enterobacteriaceae, coliforms, Pseudomonadaceae, and yeasts respectively, and CE_ct being characterized by counts always <1 Log cfu g^−1^.

The microbial viable counts of raw milk and cheese samples collected during ripening are shown in Table 2 and Table 3, for R1 and R2 samples, respectively. As a general trend, a high similarity was seen in the viable counts of control and experimental cheeses (CF_C and CF_Ot_st in R1, CF_C and CF_Ot_ct in R2, respectively). In both rounds, a similar evolution over time was observed for total mesophilic aerobes, presumptive lactococci, and presumptive thermophilic cocci, with a significant increase, of about 3 Log, in the first two days, and a further increase to reach the maximum load between 5 and 15 days, followed by a slight reduction in the last stage of maturation. The same behavior was also observed for lactobacilli in the second round (R2), while in the first round (R1), the growth of this microbial group was slower, and the maximum load was reached after 30 days of ripening in both control (CF_C) and experimental (CF_Ot_st) cheeses. As for Enterobacteriaceae, no significant differences were found between control and experimental cheeses: the maximum loads, reached after 5 days of ripening (counts in the range of 7.35–7.99 Log cfu g^−1^), were followed by a reduction, up to 4 and 2 Logs in R1 and R2, respectively; a similar trend was also observed for coliforms. *Escherichia coli* was detected in both milk batches (2.18 ± 0.00 vs. 2.53 ± 0.83 Log cfu g^−1^ in R1_M and R2_M, respectively). Regarding R1, the counts of *E. coli* in cheese samples remained almost stable during maturation, with mean values of 3.82 and 4.07 Log cfu g^−1^ in CF_C and CF_Ot_st, respectively; in R2 cheeses, maximum counts of 6.75 ± 0.22 (CF_C) and 7.16 ± 0.21 (CF_Ot_ct) Log cfu g^−1^ were found after 5 days, followed by a slow decrease. For R2, after 30 days of ripening, a lower Pseudomonadaceae count was detected in cheeses coagulated with CE_st than those coagulated with commercial rennet (5.09 ± 0.69 vs. 6.51 ± 0.20 Log cfu g^−1^, respectively), although at the end of ripening (60 days), no more differences could be evidenced. For viable counts of molds, the highest loads were found between 5 and 15 days of ripening in both cheesemaking rounds; moreover, no significant differences were observed between control and experimental cheeses, except for CF_C and CF_Ot_st analyzed after 45 days of ripening. Finally, as for yeasts, in both rounds the maximum loads were found at the fifth day of maturation, and significant differences between control and experimental cheeses were observed only in cheeses at 15 days of maturation in R2 (6.16 ± 0.63 vs. 5.09 ± 0.19 Log cfu g^−1^ in CF_C and CF_Ot_ct, respectively) and 60 days in R1 (3.54 ± 0.10 vs. 4.80 ± 0.79 Log cfu g^−1^ in CF_C and CF_Ot_st, respectively).

### 3.2. Illumina Sequencing

**Figure 4:**
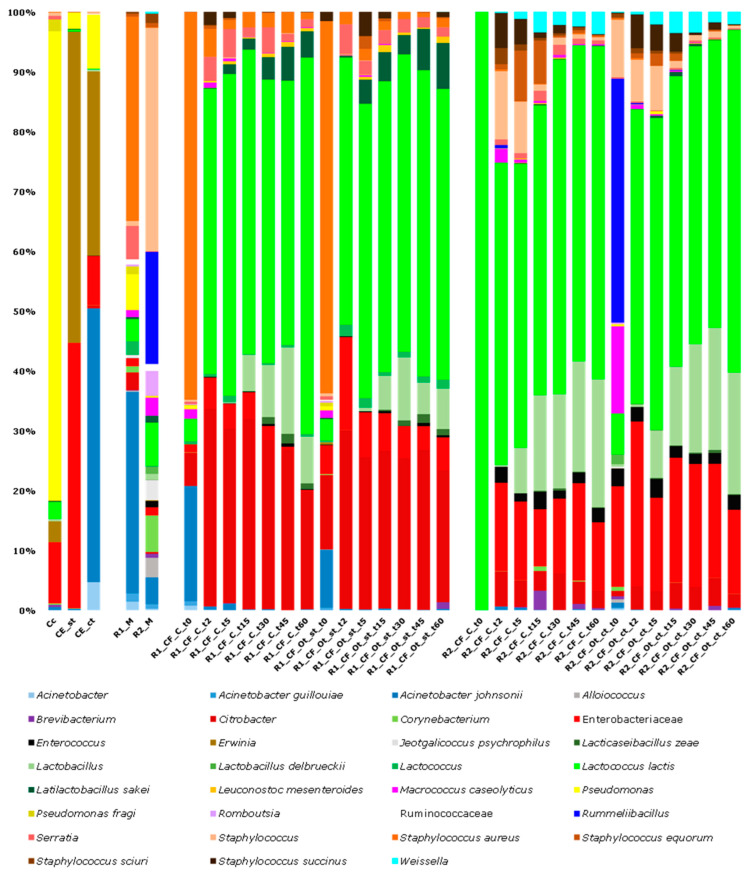
Relative frequency of the bacterial ASVs detected by Illumina DNA sequencing in thistle-based coagulants (Cc, CE_st, CE_ct), raw milk (R1_M, R2_M), control (CF_C), and experimental (CF_Ot_st and CF_Ot_ct) cheeses sampled during ripening. Only ASVs with an incidence above 1% in at least two samples are shown.

Overall, 140,388 high quality reads were used for the metataxonomic analysis of the bacterial biota, with an average of 4011 sequence per sample and a coverage > 96%. The Alpha diversity analysis showed different levels of complexity depending on the type of sample: the bacterial composition of raw milk and thistle-based coagulants highlighted a higher complexity when compared to cheeses (*p*-value < 0.05) (Supplementary Figure S2). The results from sequencing, expressed as relative frequency of bacterial ASVs, are shown in Figure 4. Milk coagulants Cc, CE_st, and CE_ct displayed the common presence of Enterobacteriaceae (6.64, 43.18, and 6.56% of the relative frequency, respectively), *Erwinia* (Cc: 2.26, CE_st: 50.65, and CE_ct: 24.58%), and *Pseudomonas* (Cc: 50.89%; CE_st: 2.67%; and CE_ct: 7.22%). In addition, CE_st showed the presence of *Lactococcus lactis* (1.87%) and *Pseudomonas fragi* (1.28%), whereas in CE_ct, *Acinetobacter* (3.75%) and *Acinetobacter johnsonii* (36.50%) were also found. Raw milk used in the two cheesemaking rounds, being R1_M and R2_M, showed the presence of *Lactococcus lactis* (3.03 and 5.62% of the relative frequency, respectively), and *Acinetobacter johnsonii* (27.56 and 3.49% of the relative frequencies, respectively). Moreover, in R1_M, *Staphylococcus aureus* (27.96%), followed by *Pseudomonas* (4.91%), and *Serratia* (4.56%), were also found, whereas R2_M included *Staphylococcus* (29.01%), *Rummeliibacillus* (14.58%), and *Corynebacterium* (4.74%).

**Figure 5:**
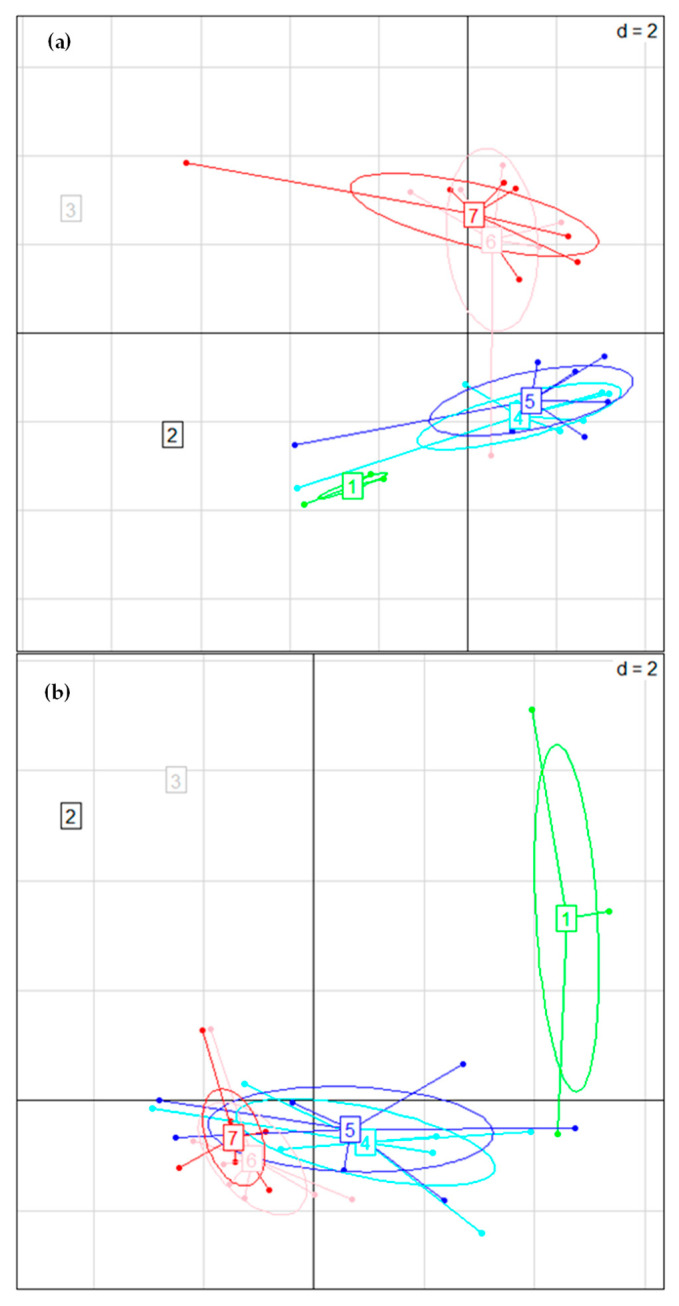
Principal component analysis (PCA) based on amplicon sequence variant (ASV) relative frequencies of thistle-based coagulants, raw milk, and Caciofiore cheeses for bacterial biota (panel (**a**)) and mycobiota (panel (**b**)). Samples are coded as follows: 1-green, coagulants; 2-black, milk used in round 1 (R1_M); 3-gray, milk used in round 2 (R2_M); 4-cyan, cheese manufactured with commercial coagulant (Cc) in R1; 5-blue, cheese manufactured with coagulant obtained from spontaneous *O. tauricum* flowers (CE_st) in R1; 6-pink, cheese manufactured with commercial coagulant (Cc) in round 2; 7-red, cheese manufactured with coagulant obtained from *O. tauricum* cultivated flowers (CE_st) in R2.

In the first round (R1), the comparison between control and experimental cheeses (CF_C vs CF_Ot_st) showed no significant differences, as depicted in the PCA plot (Figure 5, panel (a)). In fact, all cheeses were characterized by a substantial increase of *Lactococcus lactis* over time, with initial relative frequencies of 3.71 (R1_CF_C_t0) and 3.41% (R1_CF_Ot_st_t0), and relative frequencies at the end of the ripening period of 62.54 (R1_CF_C_t60) and 48.15% (R1_CF_Ot_st_t60), respectively. After five days of ripening, *Lactobacillus* spp. and *Latilactobacillus sakei* displayed a progressive increase, reaching up to 7.77 and 4.37% of the relative frequency in R1_CF_C_t60, and 6.66 and 7.61% of the relative frequency in R1_CF_Ot_st_t60, respectively. As expected, *Staphylococcus aureus* and *Acinetobacter johnsonii* were detected only at the beginning of the cheesemaking processes. *Citrobacter*, *Serratia*, and Enterobacteriaceae were stably detected at all sampling times in both production rounds, with overall mean relative frequencies of 24.08, 4.65 and 2.32%, respectively.

Even in the second round (R2), no significant differences emerged by comparing control and experimental cheeses (CF_C vs CF_Ot_ct), as shown in the PCA plot (Figure 5, panel (a)). R2_CF_C_t0 was characterized by a low bacterial diversity, with *Lactococcus lactis* being the sole species detected among bacteria, whereas in R2_CF_Ot_ct_t0 *Rummeliibacillus* ssp. and *Macrococcus caseolyticus* were detected, with 38.68 and 13.79% of the relative frequency, respectively. In both cheese manufactures, *Lactococcus lactis* drove the fermentation processes over time, reaching final relative frequencies of 54.38 and 56.57%, in R2_CF_C_t60 and R2_CF_Ot_ct_t60, respectively. Since day 5, a gradual increase was also seen for *Lactobacillus* spp., which reached final relative frequencies of 20.85 and 20.03% in R2_CF_C_t60 and R2_CF_Ot_ct_t60, respectively. *Staphylococcus* spp. was among the prevalent taxa in both cheese manufactures during the early maturation, with relative frequencies of 8.06 and 7.28% in R2_CF_C_t5 and R2_CF_Ot_ct_t5, respectively; as expected, this genus decreased below 1% at the end of ripening. Enterobacteriaceae, *Citrobacter*, *Enterococcus*, and *Weissella* were stably detected at all sampling times irrespectively of the milk coagulant used, with overall mean relative frequencies of 14.75, 3.45, 2.02, and 1.87%, respectively.

**Figure 6:**
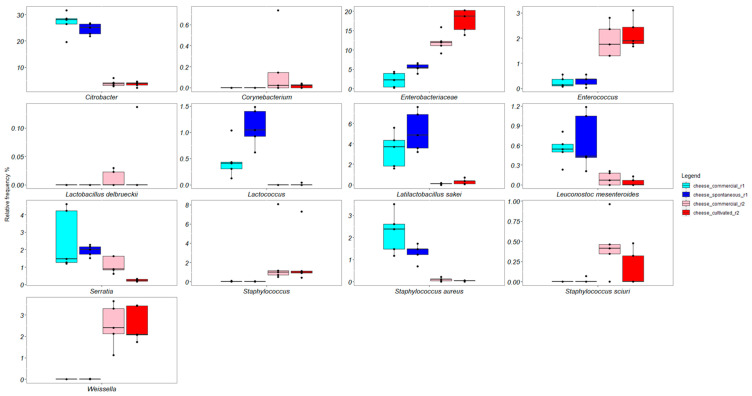
Boxplots showing relative frequencies of bacterial differential ASVs evaluated through the Kruskal–Wallis test (*p*-value < 0.05) in experimental Caciofiore cheeses.

Globally, the Kruskal–Wallis test evidenced thirteen statistically different bacterial groups among the cheese manufactures, and their boxplots are showed in Figure 6. *Citrobacter*, *Lactococcus*, *Latilactobacillus sakei*, *Leuconostoc mesenteroides*, *Serratia*, and *Staphylococcus aureus* were mainly associated with cheeses produced in round 1. Conversely, *Corynebacterium*, Enterobacteriaceae, *Enterococcus*, *Lactobacillus delbrueckii*, *Staphylococcus*, *Staphylococcus sciuri*, and *Weissella* were mainly associated with cheeses produced in round 2.

**Figure 7:**
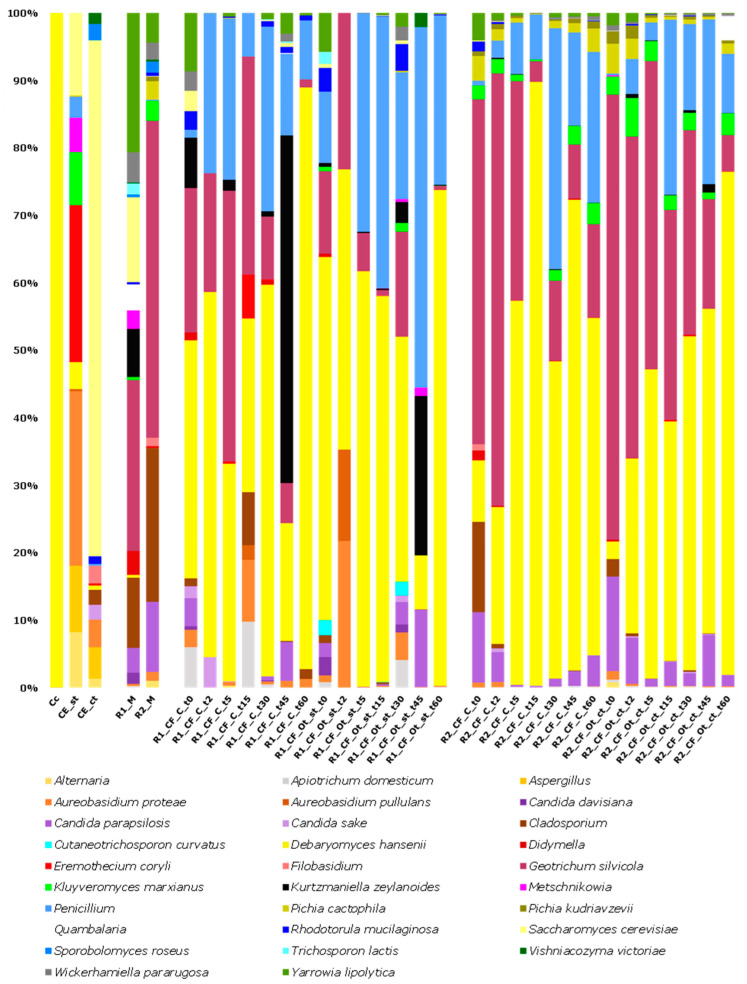
Relative frequency of the fungal ASVs detected by Illumina DNA sequencing in thistle-based coagulants (Cc, CE_st, CE_ct), milk (R1_M, R2_M), control (CF_C), and experimental (CF_Ot_st and CF_Ot_ct) cheeses sampled during ripening. Only ASVs with an incidence above 1% in at least two samples are shown.

Concerning the metataxonomic analysis of the mycobiota, 1,871,974 high quality reads were used, with an average of 53,485 sequence per sample and a coverage > 99%. The Alpha diversity analysis displayed different levels of complexity depending on the type of sample. In more detail, the fungal composition of raw milk showed a higher complexity regarding cheeses, whereas the thistle-based coagulants showed no significant differences if compared to raw milk and cheeses sampled at different maturation stages (*p*-value < 0.05) (Supplementary Figure S3). The results from Illumina DNA sequencing, expressed as relative frequency of fungal ASVs, are shown in Figure 7. The mycobiota of the commercial coagulant (Cc) was dominated by *Debaryomyces hansenii*, attesting at 99.51% of the relative frequency, whereas CE_st and CE_ct shared the presence of *Saccharomyces cerevisiae* (7.22 and 55.30% of the relative frequency, respectively), *Aerobasidium proteae* (15.24 and 2.96% of the relative frequency, respectively), *Aspergillus* (5.82 and 3.36%, respectively), *Alternaria* (4.85 and 0.96%, respectively), and *Debaryomyces hansenii* (2.36 and 0.46%, respectively). In addition, CE_st included *Eremothecium coryli*, *Kluyveromyces marxianus*, *Metschnikowia*, and *Penicillium*, with relative frequency of 13.73, 4.64, 3.01, and 1.84%, respectively. As for raw milk batches R1_M and R2_M, the common presence of *Geotrichum silvicola* (24.82 and 45.22% of the relative frequency, respectively), *Cladosporium* (10.21 and 21.93%, respectively), *Yarrowia lipolytica* (20.26 and 4.24%, respectively), *Candida parapsilosis* (3.61 and 10.01%, respectively), and *Wickerhamiella pararugosa* (4.42 and 2.44%, respectively) was highlighted. *Saccharomyces cerevisiae*, *Kurtzmaniella zeylanoides*, and *Didymella* were also detected in R1_M, with 12.35, 7.01, and 3.49% of the relative frequency, respectively, while *Kluyveromyces marxianus* and *Pichia cactophila* were identified in R2_M, with 2.87 and 2.64% of the relative frequency, respectively.

Cheeses manufactured in round 1 showed no significant differences related to the type of coagulant, as depicted in the PCA plot (Figure 5, panel (b)). *Debaryomyces hansenii* was predominant in both cheese manufactures, reaching up to 85.96 (R1_CF_C_t60) and 73.14% (R1_CF_Ot_st_t60) of the relative frequency at the end of ripening. *Penicillium* spp. was detected at variable levels in almost all the cheese samples, with an overall mean relative frequency of 19.94%. A decreasing trend was seen for *Geotrichum silvicola* in both cheese manufactures; *Yarrowia lipolytica* was among the prevalent taxa exclusively at the beginning of the monitoring process, with relative frequencies of 8.33 and 5.42% in R1_CF_C_t0 and R1_CF_Ot_st_t0, respectively. Similar results were found for the cheeses manufactured in the second round (R2); in both control and experimental cheese manufactures, *Debaryomyces hansenii* increased with time up to 49.58 (R2_CF_C_t60) and 73.97% (R2_CF_Ot_ct_t60) of the relative frequency. An analogous trend was observed for *Penicillium* spp., which after 60 days of ripening reached 22.18 (R2_CF_C_t60) and 8.72% (R2_CF_Ot_ct_t60) of the relative frequency. On the contrary, *Geotrichum silvicola* and *Candida parapsilosis* decreased over time. Like *Y. lipolytica*, even *Cladosporium* spp. was identified among the main taxa just after molding, attesting at 13.24 (R2_CF_C_t0) and 2.53% (R2_CF_Ot_ct_t0) of the relative frequency. *Pichia cactophila* was stably detected in all cheese samples, with an overall mean relative frequency of 1.64%. Again, as shown in the PCA plot (Figure 5, panel (b)), no significant differences emerged by comparing control (R2_CF_C) and experimental (R2_CF_Ot_ct) cheeses.

**Figure 8:**
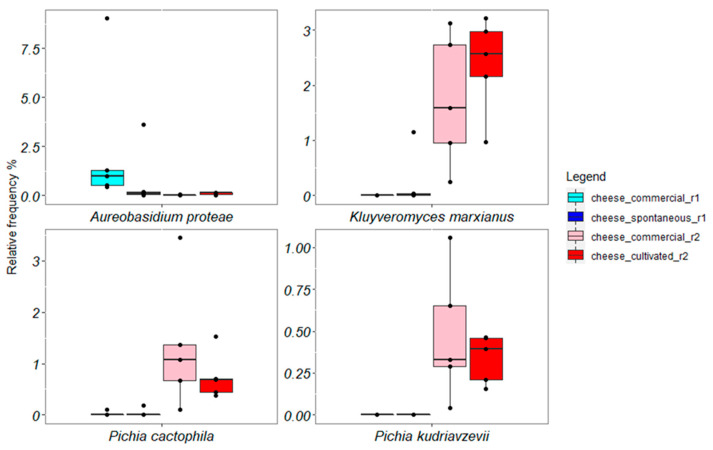
Boxplots showing relative frequencies of mycobiota differential ASVs evaluated through the Kruskal–Wallis test (*p*-value < 0.05) in experimental Caciofiore cheeses.

Globally, the Kruskal–Wallis test evidenced four statistically different fungal groups among cheese manufactures, and their boxplots are showed in Figure 8. *Aerobasidium proteae* was mainly associated with Caciofiore cheeses produced in round 1, whereas *Kluyveromyces marxianus*, *Pichia cactophila*, and *Pichia kudriavzevii* with Caciofiore cheeses produced in round 2.

## 4. Discussion

To the author’s knowledge, this is the very first report describing the exploitation of *O. tauricum* Willd. for cheese manufacturing. The present investigation is also among the very few ones till date carried out to evaluate the impact of thistle rennet on cheese microbial dynamics during fermentation and ripening. From a microbiological point of view, as a main ingredient of thistle curdled cheeses, thistle rennet must not represent a source of undesirable microorganisms, with detrimental effects on cheeses (e.g., off-odors or off-flavors, undesired changes in texture, appearance, or palatability, etc.) or consumers’ health (e.g., foodborne disease, food poisoning). By contrast, the occurrence of pro-technological or functional microbes in thistle rennet might be advantageous for obtaining high-quality cheeses, especially when primary or secondary starters are not used, as in the cheeses herein manufactured.

### 4.1. Viable Counting

Except for presumptive lactobacilli, viable counts of the coagulant obtained from spontaneous *O. tauricum* populations (CE_st) were consistent with those previously reported for crude extracts of other thistle species with a documented milk-coagulating potential [33,34,35,44]. As a general trend, counts of CE_ct were significantly lower than those of the extract from spontaneous *O. tauricum* (CE_st). For Enterobacteriaceae, coliforms, and Pseudomonadaceae, viable counts of CE_ct < 1 Log cfu g^−1^ suggest a higher hygienic quality of these extracts compared to those prepared from spontaneous *O. tauricum* (CE_st); this finding might be tentatively ascribed to the differences in the management of cultivated fields than pastures or fallow lands, more often accessed or grazed by wild animals or even livestock, whose feces are known to harbor the aforementioned microorganisms [45,46]. In this regard, recent comparative metagenomic analyses also highlighted the strong influence of farming practices on taxonomic and functional diversity of phyllosphere microbes [47].

However, both control and experimental cheeses herein manufactured were dominated by lactic acid bacteria, which exhibited an intense growth in the early stage of maturation (0–15 days), associated with a significant pH drop, followed by a stabilization phase (30–60 days). Viable counts of presumptive lactococci and thermophilic cocci in raw milk and cheeses during their early maturation were comparable to those overall collected by Cardinali et al. [34] in the same specialty cheese manufactured with a crude extract of *Carlina acanthifolia* All. subsp. *acanthifolia*.

Enterobacteriaceae, coliforms, and *E. coli* were enumerated in raw milk used in R1 and R2, respectively. In cheeses, after five days of maturation, they decreased, but without disappearing completely up until the end of maturation (60 days), when higher counts were seen in R2 than R1. Members of the Enterobacteriaceae family have previously been detected in raw milk and raw milk cheeses, especially in the early cheese maturation [48,49,50]. The presence of these microorganisms in cheese is controversial for safety and technological issues [51]: in fact, though some species are known to produce aromatic volatile compounds [52], some others include strains with pathogenic traits [53].

Regarding eumycetes, the differences found in the viable counts of yeasts and molds in the three assayed thistle coagulants did not affect the fungal dynamics during cheese ripening. Similar evidence emerged in the study carried out by Cardinali et al. [35].

### 4.2. Illumina Sequencing

Over the past decades, to overcome the limitations of conventional culture-dependent analyses, DNA-based techniques have gained increasing acceptance, allowing for better investigation of the microbiota of foods [54,55,56]. Among these techniques, next-generation sequencing has previously been exploited for the investigation of complex microbial communities of thistle-curdled cheeses [13,14,15]. In the present study, the application of Illumina sequencing on nucleic acids extracted from thistle-based coagulants, raw milk, and cheese samples provided a comprehensive insight on the dynamics of bacteria and fungi, from the raw ingredients to mature cheeses.

Cc, CE_st, and CE_ct were mainly dominated by spoilage and environmental microorganisms, whereas lactic acid bacteria were only found at low relative frequencies, with *Lactococcus lactis* being detected in Cc, CE_st, and CE_ct, *Latilactobacillus sakei* in Cc and CE_st, and *Lactobacillus* spp. in Cc and CE_ct, respectively.

*Acinetobacter johnsonii*, *Staphylococcus* spp., *Geotrichum silvicola*, *Cladosporium* spp., *Yarrowia lipolytica*, *Candida parapsilosis*, and *Wickerhamiella pararugosa* were among the prevalent taxa in both batches of Sopravissana ewe’s raw milk. The occurrence of *Acinetobacter johnsonii*, *Staphylococcus* spp., and *Candida parapsilosis* has previously been documented in raw milk of the same sheep breed [34,35]. *Geotrichum* species, mainly *G. candidum*, are also commonly associated with milk and cheese [57,58,59]; although recognized as synonym of *Galactomyces candidus* [60] due to phylogenetic affinity, to the best of the authors’ knowledge this is the first study reporting the identification of *Geotrichum silvicola* in raw ewe’s milk and cheese. Great interest is addressed to the yeast *Yarrowia lipolytica* for its proteolytic and lipolytic activities, and the consequent aroma development during cheese ripening [61,62,63]. To date, *Y. lipolytica* has frequently been identified in cheeses [64], whereas its occurrence in milk has more poorly been documented [65,66,67,68]. However, Corbo et al. [69] remarked the predominance of *Y. lipolytica* in ewe’s milk, which is characterized by a higher fat content than cow’s and goat’s milk. As for *Wickerhamiella pararugosa* (synonym *Candida pararugosa*), this yeast has recently been found among the minority species in various dairy products [13,70,71] and milk [72]. Air is considered the main contamination source of *Cladosporium* spp. [73,74], a mold commonly identified in raw milk [75,76,77].

Several minority bacterial and fungal taxa were also identified in the two raw milk batches by Illumina DNA sequencing; though *Latilactobacillus sakei*, *Enterococcus* spp., *Macrococcus caseolyticus*, *Lactobacillus* spp., and *Kluyveromyces marxianus* were detected in both R1_M and R2_M, these batches differed significantly in number and relative abundance of the detected taxa, with R1_M harboring *Kurtzmaniella zeylanoides*, and *Saccharomyces cerevisiae* and R2_M being characterized by the occurrence of *Lactobacillus delbrueckii*, *Weissella* spp., and *Pichia* spp. As previously suggested by other authors, the differences emerged between the two raw milk samples, collected in spring (R1_M) and late summer (R2_M) 2021, respectively, might be likely ascribed to the seasonality [78,79,80].

The microbial composition of cheeses, and especially of those analyzed immediately after molding, was clearly affected by the microbial composition of the raw milk, irrespective of the thistle coagulant employed in cheesemaking. A plethora of taxa were detected in samples collected throughout the two production rounds, being *Acinetobacter johnsonii*, *Citrobacter* spp., *Staphylococcus aureus*, *Lactococcus lactis*, *Serratia* spp., *Debaryomyces hansenii*, *Geotrichum silvicola*, *Kurtzmaniella zeylanoides*, and *Yarrowia lipolytica* in R1, and Enterobacteriaceae, *Lactococcus lactis*, *Macrococcus caseolyticus*, *Enterococcus* spp., *Weissella* spp., *Rummeliibacillus* spp., *Staphylococcus* spp., *Candida parapsilosis*, *Cladosporium* spp., *Debaryomyces hansenii*, *Geotrichum silvicola*, *Kluyveromyces marxianus*, and *Pichia cactophila* in R2.

However, in both production rounds, *Lactococcus lactis* among bacteria and *Debaryomyces hansenii* among eumycetes dominated the microbial dynamics during fermentation and maturation of both control and experimental cheeses. The latter finding agrees with those of previous studies carried out in typical Mediterranean sheep and goat cheeses [81,82,83,84,85,86,87]. *L. lactis* is a homofermentative starter microorganism known for its ability to lower the pH and drive cheese fermentation [88]. *D. hansenii* is a yeast species that plays a key role during cheese ripening through the production of proteolytic and lipolytic enzymes acting on milk proteins and fat [89].

Furthermore, concerning bacterial dynamics, in both control and experimental cheeses, an increase in the relative frequency of *Lactobacillus* spp. was detected starting from t_15_ in R1, and t_5_ in R2, respectively, until the last sampling time (t_60_), thus showing the ability of this genus to adapt to the cheese habitat, and suggesting its important role during cheese fermentation and ripening [90]. A similar behavior has been also observed for *Latilactobacillus sakei* in R1, and *Weissella* spp. in R2. All the cited lactic acid bacteria have previously been found as part of the bacterial biota of raw milk cheeses [13,91,92,93]. As for fungal evolution, in R1, most of the predominant taxa in milk and early ripened cheeses were found as minority components at the end of maturation. In R2, *Candida parapsilosis* and *Geotrichum silvicola* were among the dominant species until the end of maturation despite a significant reduction over time. In the same production round, *Kluyveromyces marxianus* was also detected; the occurrence of this yeast species in ewe’s milk cheeses is well documented, where it contributes to maturation and aroma formation owing to its ability to metabolize lactose, proteins, and fat [81,83,84,94].

For the stable detection of the genus *Penicillium* in all cheeses herein analyzed, independent of the production round and the rennet used, it might be likely explained by a contamination from the air or the ripening environment, as previously been suggested for other cheeses [74].

Viable counts also showed a stable occurrence of presumptive Pseudomonadaceae in samples collected from both production rounds; however, neither *Pseudomonas* spp. nor *Pseudomonas fragi* were detected in any cheese sample by Illumina sequencing after 30 days of maturation, thus once more underlying the importance of using a combined analytical approach, based on both culture-dependent and -independent analyses.

## 5. Conclusions

The combination of conventional microbiological analyses and Illumina DNA sequencing applied on experimental and commercial thistle-based coagulants, raw milk, and cheeses sampled at different ripening times allowed an in-depth investigation of the microbial dynamics occurring during fermentation and ripening of Caciofiore cheese manufactured with raw ewe’s milk curdled with thistle rennet. Despite the differences that emerged between the three milk coagulants, the results herein collected revealed that the microbiological quality of raw milk is among the main factors affecting the evolution of the bacterial and fungal biota during cheese fermentation and ripening. By contrast, in accordance with the results previously collected by Aquilanti et al. [33] and Cardinali et al. [34,35], a neglectable impact of thistle rennet on cheese microbial dynamics emerged, with the rennet microbiota being mainly dominated by adventitious microorganisms with neither dairy attitude nor detrimental traits. In addition, from a microbiological point of view, at the end of ripening, mature cheeses manufactured with the experimental coagulants were equivalent to those produced with a commercially available thistle rennet obtained from *C. cardunculus*, thus supporting the potential use of the newly explored rennet from *O. tauricum* for manufacturing of ewe’s milk cheeses. In this regard, the evidence in present study supports the large-scale cultivation of *O. tauricum* for production of a new alternative thistle rennet to be proposed to dairy farmers for manufacturing of Mediterranean ovine cheeses.

## Figures and Tables

**Table 1:** Viable counts of thistle coagulants. Cc: commercial milk coagulant; CE_st: extract from spontaneous *O. tauricum* flowers; CE_ct: extract from cultivated *O. tauricum* flowers.

Microbial Group	Cc	CE_st	CE_ct
Total mesophilic aerobes	<1 ^c^	4.91 ± 0.03 ^a^	3.55 ± 0.10 ^b^
Presumptive lactobacilli	<1 ^a^	<1 ^a^	<1 ^a^
Presumptive lactococci	<1 ^c^	3.96 ± 0.06 ^a^	2.91 ± 0.14 ^b^
Presumptive thermophilic cocci	<1 ^c^	3.42 ± 0.04 ^a^	2.51 ± 0.03 ^b^
Enterobacteriaceae	<1 ^b^	3.04 ± 0.06 ^a^	<1 ^b^
Coliforms	<1 ^b^	3.64 ± 0.16 ^a^	<1 ^b^
*Escherichia coli*	<1 ^a^	<1 ^a^	<1 ^a^
Pseudomonadaceae	<1 ^b^	3.64 ± 0.11 ^a^	<1 ^b^
Molds	<1 ^b^	3.33 ± 0.00 ^a^	3.07 ± 0.00 ^a^
Yeasts	6.36 ± 0.02 ^a^	2.90 ± 0.00 ^b^	<1 ^c^

Values are expressed as Log cfu g^−1^ ± standard deviation. Within each row, overall means with different superscript letters are significantly different (*p* < 0.05).

**Table 2:** Viable counts of milk used in the first cheesemaking round (R1_M), and control (CF_C), and experimental (CF_Ot_st) Caciofiore cheeses sampled during ripening.

Microbial Group	Sample Code	Milk	Cheese						
			t_0_	t_1_	t_2_	t_3_	t_4_	t_5_	t_6_
Total mesophilic aerobes	CF_C	5.79 ± 0.20	6.00 ± 0.83	8.68 ± 0.28	9.28 ± 0.04	9.06 ± 0.07	9.00 ± 0.16	8.51 ± 0.39	8.72 ± 0.61
	CF_Ot_st		6.18 ± 0.69	8.64 ± 0.29	9.21 ± 0.11	9.06 ± 0.06	9.12 ± 0.02	8.75 ± 0.21	8.63 ± 0.20
Presumptive lactobacilli	CF_C	1.56 ± 1.80	1.46 ± 1.47	3.42 ± 0.90	5.41 ± 0.89	7.53 ± 0.91	8.94 ± 0.15	8.38 ± 0.42	8.51 ± 0.39
	CF_Ot_st		1.54 ± 1.78	3.37 ± 1.17	5.55 ± 0.69	7.28 ± 0.77	8.83 ± 0.13	8.40 ± 0.36	8.27 ± 0.08
Presumptive lactococci	CF_C	4.75 ± 0.17	5.04 ± 0.20	8.31 ± 0.70	9.24 ± 0.03	9.02 ± 0.08	8.95 ± 0.17	8.30 ± 0.53	8.64 ± 0.60
	CF_Ot_st		5.08 ± 0.18	8.46 ± 0.68	8.57 ± 0.89	9.07 ± 0.10	8.99 ± 0.05	8.56 ± 0.12	8.46 ± 0.18
Presumptive thermophilic cocci	CF_C	4.76 ± 0.15	5.01 ± 0.08	8.53 ± 0.39	8.88 ± 0.28	8.85 ± 0.20	8.59 ± 0.24	7.31 ± 0.29	6.88 ± 0.23
	CF_Ot_st		4.98 ± 0.09	8.19 ± 0.75	8.73 ± 0.36	8.78 ± 0.20	8.57 ± 0.35	7.70 ± 0.16	7.00 ± 0.22
Enterobacteriaceae	CF_C	4.12 ± 0.20	4.62 ± 0.22	7.69 ± 0.58	7.95 ± 0.32	6.62 ± 0.44	5.38 ± 0.66	4.36 ± 0.61	3.32 ± 1.53
	CF_Ot_st		4.83 ± 0.35	7.71 ± 0.45	7.99 ± 0.17	6.56 ± 0.49	4.76 ± 0.92	4.11 ± 0.43	3.46 ± 1.11
Coliforms	CF_C	4.26 ± 0.31	4.71 ± 0.09 ^b^	7.69 ± 0.56	7.83 ± 0.40	7.70 ± 0.09	5.48 ± 0.74	3.43 ± 1.66	3.49 ± 1.38
	CF_Ot_st		5.26 ± 0.15 ^a^	7.87 ± 0.37	7.97 ± 0.56	7.65 ± 0.14	4.87 ± 1.08	4.04 ± 0.81	3.81 ± 1.30
*Escherichia coli*	CF_C	2.18 ± 0.00	2.62 ± 0.06	4.18 ± 0.63	4.38 ± 0.96	4.55 ± 1.17	4.15 ± 1.07	3.81 ± 0.87	3.08 ± 1.43
	CF_Ot_st		2.47 ± 0.75	4.71 ± 0.35	4.85 ± 0.26	5.06 ± 0.63	3.75 ± 1.11	4.25 ± 0.53	3.38 ± 0.95
Pseudomonadaceae	CF_C	4.77 ± 0.32	4.03 ± 0.51	6.42 ± 0.42	7.09 ± 0.29	7.19 ± 0.13	6.51 ± 0.20 ^a^	4.96 ± 0.58	4.52 ± 0.70
	CF_Ot_st		4.29 ± 0.80	6.37 ± 0.39	7.12 ± 0.21	7.38 ± 0.35	5.09 ± 0.69 ^b^	4.73 ± 0.98	3.99 ± 0.87
Molds	CF_C	2.10 ± 0.14	2.31 ± 0.01	2.73 ± 0.09	4.13 ± 0.76	3.09 ± 0.14	2.78 ± 0.11	2.16 ± 0.36 ^b^	2.77 ± 0.68
	CF_Ot_st		2.88 ± 0.58	2.35 ± 0.26	3.78 ± 0.38	3.74 ± 0.56	2.23 ± 0.15	3.86 ± 0.49 ^a^	3.07 ± 0.53
Yeasts	CF_C	3.03 ± 0.57	2.99 ± 0.08	3.96 ± 0.04	5.70 ± 0.60	4.14 ± 1.17	3.90 ± 1.51	3.89 ± 0.67	3.54 ± 0.10 ^b^
	CF_Ot_st		3.57 ± 1.22	3.35 ± 0.38	6.16 ± 0.56	4.85 ± 1.69	3.15 ± 0.47	3.82 ± 0.91	4.80 ± 0.79 ^a^

Values are expressed as Log cfu mL^−1^ or g^−1^ ± standard deviation. Different superscript letters on the same main row (microbial group) at the same time of ripening indicate significant differences (*p* < 0.05) between CF_C and CF_Ot_st. t_0_: cheeses analyzed immediately after molding; t_1_: 2-day-ripened cheeses; t_2_: 5-day-ripened cheeses; t_3_: 15-day-ripened cheeses; t_4_: 30-day-ripened cheeses; t_5_: 45-day-ripened cheeses; t_6_: 60-day-ripened cheeses.

**Table 3:** Viable counts of milk used in the second cheesemaking round (R2_M), and control (CF_C), and experimental (CF_Ot_ct) Caciofiore cheeses sampled during ripening.

Microbial Group	Sample Code	Milk	Cheese						
			t_0_	t_1_	t_2_	t_3_	t_4_	t_5_	t_6_
Total mesophilic aerobes	CF_C	5.26 ± 0.45	5.41 ± 0.12	8.97 ± 0.07	9.15 ± 0.29	9.05 ± 0.20	8.53 ± 0.48	8.94 ± 0.23	8.42 ± 0.12
	CF_Ot_ct		5.41 ± 0.16	8.83 ± 0.10	8.98 ± 0.07	8.98 ± 0.05	8.60 ± 0.24	8.69 ± 0.11	8.12 ± 0.13
Presumptive lactobacilli	CF_C	4.76 ± 0.97	5.16 ± 0.29	8.20 ± 0.07	9.15 ± 0.21	9.01 ± 0.27	8.55 ± 0.53	8.57 ± 0.28	8.49 ± 0.27
	CF_Ot_ct		4.89 ± 0.34	8.12 ± 0.16	8.97 ± 0.05	8.85 ± 0.04	8.59 ± 0.18	8.60 ± 0.06	8.26 ± 0.04
Presumptive lactococci	CF_C	5.05 ± 0.71	5.27 ± 0.29	9.09 ± 0.17	9.19 ± 0.15	9.06 ± 0.19	8.64 ± 0.51	8.37 ± 0.24	8.19 ± 0.19
	CF_Ot_ct		5.22 ± 0.30	8.79 ± 0.20	9.13 ± 0.07	8.84 ± 0.06	8.07 ± 0.50	8.21 ± 0.07	7.81 ± 0.17
Presumptive thermophilic cocci	CF_C	4.94 ± 0.94	5.29 ± 0.28	9.02 ± 0.14	9.20 ± 0.18	8.87 ± 0.09	8.30 ± 0.55	8.33 ± 0.36	8.16 ± 0.15
	CF_Ot_ct		5.96 ± 1.30	8.83 ± 0.21	9.11 ± 0.17	8.78 ± 0.22	7.98 ± 0.50	8.01 ± 0.55	7.70 ± 0.15
Enterobacteriaceae	CF_C	3.16 ± 0.47	4.31 ± 0.33	7.36 ± 0.14	7.35 ± 0.16	6.90 ± 0.11	6.11 ± 0.17	6.06 ± 0.54	5.53 ± 0.68
	CF_Ot_ct		3.53 ± 1.38	7.64 ± 0.09	7.67 ± 0.16	7.16 ± 0.09	6.52 ± 0.24	6.47 ± 0.67	5.50 ± 0.42
Coliforms	CF_C	3.41 ± 0.78	3.53 ± 1.09	7.35 ± 0.23	7.23 ± 0.26	6.50 ± 0.51	5.90 ± 0.10	5.62 ± 0.09	4.83 ± 0.05
	CF_Ot_ct		4.28 ± 0.22	7.86 ± 0.60	7.35 ± 0.46	6.52 ± 0.65	6.08 ± 0.84	5.57 ± 0.11	4.48 ± 0.00
*Escherichia coli*	CF_C	2.53 ± 0.83	3.37 ± 0.52	6.68 ± 0.20	6.75 ± 0.22	6.51 ± 0.25	6.32 ± 0.30	6.17 ± 0.83	5.77 ± 0.57
	CF_Ot_ct		3.61 ± 0.53	7.07 ± 0.52	7.16 ± 0.21	6.70 ± 0.21	6.68 ± 0.12	6.38 ± 0.33	5.79 ± 0.19
Pseudomonadaceae	CF_C	3.85 ± 0.25	3.75 ± 0.12	6.59 ± 0.36	6.13 ± 0.18	6.13 ± 0.45	3.58 ± 0.50	3.29 ± 0.13	2.77 ± 0.14
	CF_Ot_ct		3.82 ± 0.06	6.88 ± 0.08	6.30 ± 0.12	6.23 ± 0.28	3.97 ± 1.12	3.63 ± 0.83	2.11 ± 0.00
Molds	CF_C	3.08 ± 0.47	2.81 ± 0.38	2.92 ± 0.11	5.43 ± 0.73	3.51 ± 1.18	2.93 ± 1.03	2.12 ± 0.49	2.50 ± 0.06
	CF_Ot_ct		2.75 ± 0.32	3.03 ± 0.03	5.31 ± 0.39	4.47 ± 0.11	2.89 ± 0.89	3.19 ± 0.72	2.49 ± 0.13
Yeasts	CF_C	3.63 ± 0.28	3.34 ± 0.31	4.49 ± 0.02	6.72 ± 0.87	6.16 ± 0.63 ^a^	4.77 ± 0.55	4.88 ± 0.36	4.48 ± 0.35
	CF_Ot_ct		3.42 ± 0.18	4.24 ± 0.31	5.84 ± 0.73	5.09 ± 0.19 ^b^	4.55 ± 0.22	5.46 ± 0.34	4.55 ± 0.37

Values are expressed as Log cfu mL^−1^ or g^−1^ ± standard deviation. Different superscript letters on the same main row (microbial group) at the same time of ripening indicate significant differences (*p* < 0.05) between CF_C and CF_Ot_ct. t_0_: cheeses analyzed immediately after molding; t_1_: 2-day-ripened cheeses; t_2_: 5-day-ripened cheeses; t_3_: 15-day-ripened cheeses; t_4_: 30-day-ripened cheeses; t_5_: 45-day-ripened cheeses; t_6_: 60-day-ripened cheeses.

## Data Availability

Data are contained within the article.

## Supplementary Materials

The following supporting information can be downloaded at: https://www.mdpi.com/article/10.3390/microorganisms11010219/s1. Figure S1: Flow chart of the cheesemaking process of Caciofiore. Figure S2: Alpha diversity plots of Pielou’s evenness and Shannon indices for bacterial biota based on the sample matrices (Caciofiore cheeses, raw milks, and thistles coagulants). Figure S3: Alpha diversity plots of Pielou’s evenness and Shannon indices for mycobiota based on the sample matrices (Caciofiore cheeses, raw milks, and thistle coagulants).
