# Supplementary material for: Microbial Dynamics of a Specialty Italian Raw Ewe’s Milk Cheese Curdled with Extracts from Spontaneous and Cultivated Onopordum tauricum Willd
Source: Microorganisms. 2023 Jan 15;11(1):219. doi: 10.3390/microorganisms11010219 (PMC9863127; doi:10.3390/microorganisms11010219)
Supplement: Supplementary file 1 [file microorganisms-11-00219-s001.zip › microorganisms-2132059-supplementary.pdf]

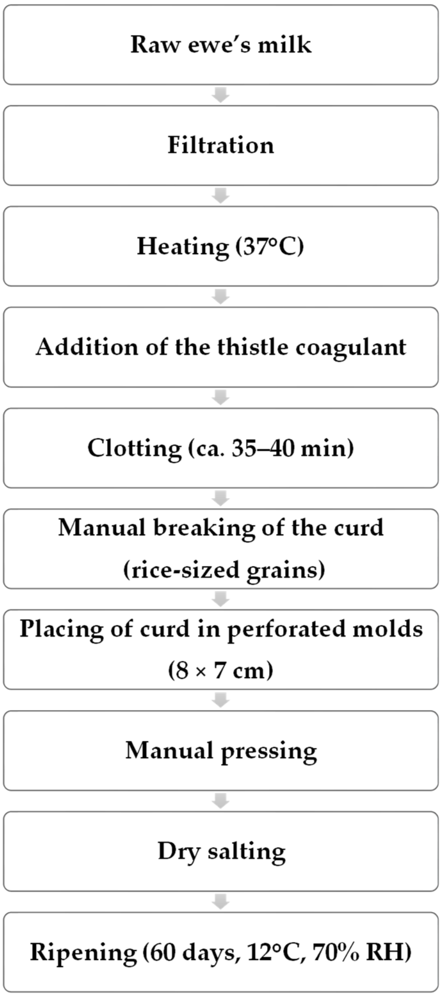

Figure S1. Flow chart of the cheesemaking process of Caciofiore.

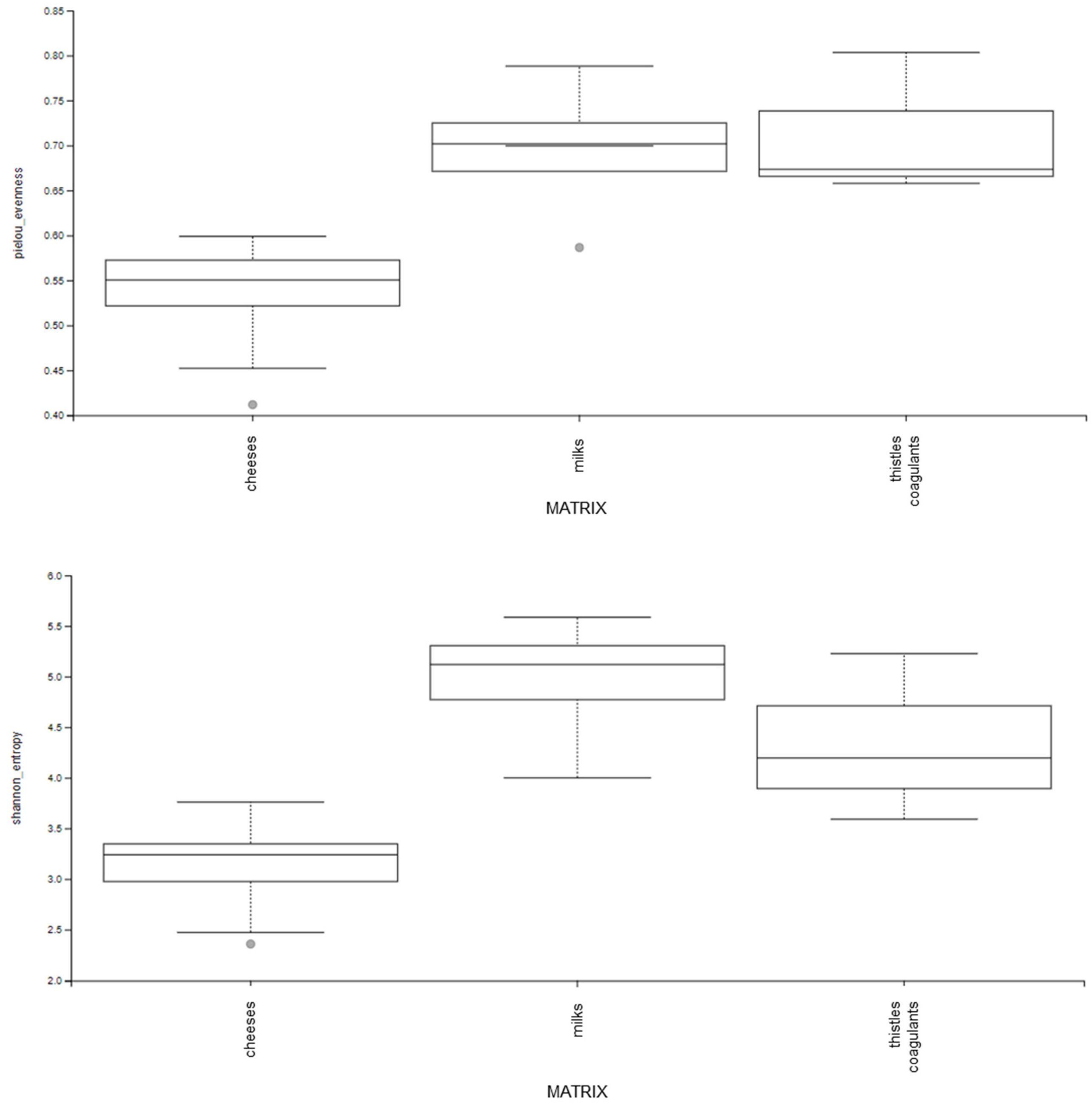

Figure S2. Alpha diversity plots of Pielou's evenness and Shannon indices for bacterial biota based on the sample matrices (Caciofiore cheeses, raw milks, and thistles coagulants). The line inside each box represents the median value. Outliers are shown as dots.

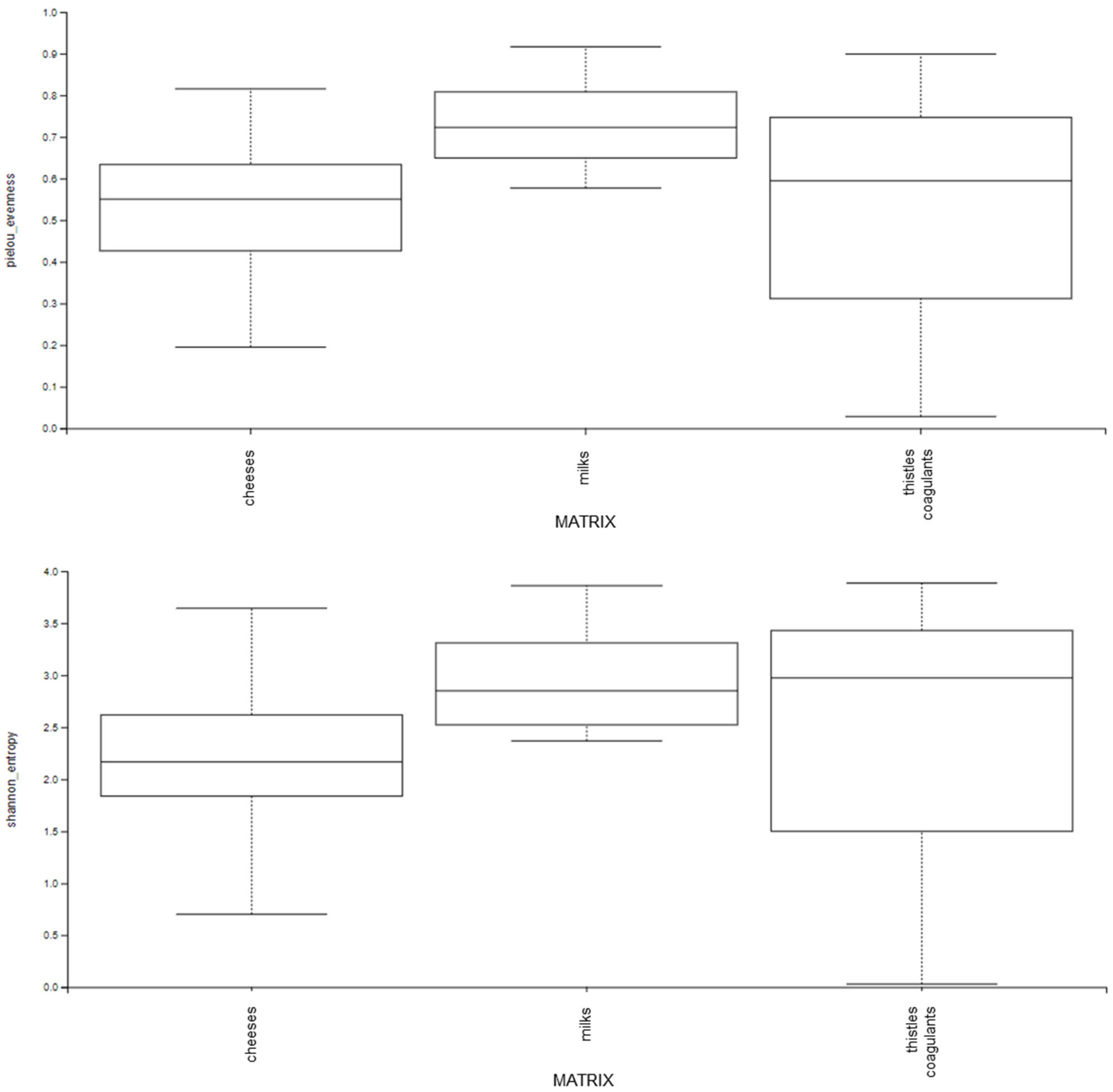

Figure S3. Alpha diversity plots of Pielou's evenness and Shannon indices for mycobiota based on the sample matrices (Caciofiore cheeses, raw milks, and thistle coagulants). The line inside each box represents the median value.
